# Long-term environmental metal exposure is associated with hypomethylation of CpG sites in *NFKB1* and other genes related to oncogenesis

**DOI:** 10.1186/s13148-023-01536-3

**Published:** 2023-08-07

**Authors:** Ani Stepanyan, Anna Petrackova, Siras Hakobyan, Jakub Savara, Suren Davitavyan, Eva Kriegova, Arsen Arakelyan

**Affiliations:** 1 Institute of Molecular Biology, National Academy of Sciences, Yerevan, Republic of Armenia; 2 Department of Immunology, Faculty of Medicine and Dentistry, Palacký University Olomouc and University Hospital Olomouc, Olomouc, Czech Republic; 3 Department of Computer Science, Faculty of Electrical Engineering and Computer Science, VSB-Technical University of Ostrava, Ostrava, Czech Republic

**Keywords:** Toxic metal, Environmental exposure, DNA methylation, *NFKB1*, *CDKN2A*, *IGF2*, *ESR1*, Uranium

## Abstract

**Background:**

Long-term environmental exposure to metals leads to epigenetic changes and may increase risks to human health. The relationship between the type and level of metal exposure and epigenetic changes in subjects exposed to high concentrations of metals in the environment is not yet clear. The aim of our study is to find the possible association of environmental long-term exposure to metals with DNA methylation changes of genes related to immune response and carcinogenesis. We investigated the association of plasma levels of 21 essential and non-essential metals detected by ICP-MS and the methylation level of 654 CpG sites located on *NFKB1, CDKN2A, ESR1, APOA5, IGF2 *and* H19* genes assessed by targeted bisulfite sequencing in a cohort of 40 subjects living near metal mining area and 40 unexposed subjects. Linear regression was conducted to find differentially methylated positions with adjustment for gender, age, BMI class, smoking and metal concentration.

**Results:**

In the metal-exposed group, five CpGs in the *NFKB1* promoter region were hypomethylated compared to unexposed group. Four differentially methylated positions (DMPs) were associated with multiple metals, two of them are located on *NFKB1* gene, and one each on *CDKN2A* gene and *ESR1* gene. Two DMPs located on *NFKB1* (chr4:102500951, associated with Be) and *IGF2* (chr11:2134198, associated with U) are associated with specific metal levels. The methylation status of the seven CpGs located on *NFKB1* (3)*, ESR1* (2) and *CDKN2A* (2) positively correlated with plasma levels of seven metals (As, Sb, Zn, Ni, U, I and Mn).

**Conclusions:**

Our study revealed methylation changes in *NFKB1, CDKN2A, IGF2* and *ESR1* genes in individuals with long-term human exposure to metals. Further studies are needed to clarify the effect of environmental metal exposure on epigenetic mechanisms and pathways involved.

**Supplementary Information:**

The online version contains supplementary material available at 10.1186/s13148-023-01536-3.

## Background

Toxic metals and metalloids are one of the most dangerous groups of environmental contaminants with adverse effects on living organisms. Because of their bioaccumulation properties, they enter the food chain and pass through trophic levels in the ecosystem. There is growing evidence that chronic exposure to essential and non-essential metals may increase the risk of cancer, renal, autoimmune, neurological, neurodegenerative, hematological and cardiovascular diseases after long-term environmental and occupational human exposure [1–3]. Binding to proteins, alteration of their structure and function and generation of reactive oxygen species, which damage lipids, proteins and DNA, are the primary focus for studies of molecular mechanisms associated with harmful metals toxicity [4–6]. Toxic metal exposure leads to oxidative/nitrosative stress and suppresses cell intrinsic antioxidant defense, which is manifested by the depletion of antioxidant enzymes, alterations of DNA methylation and DNA damage [6–9].

Oxidative stress-derived epigenetic dysregulation at gene promoters is critical for gene expression and leads to alterations in metabolic, genetic and signal transduction pathways and their functions [8, 10, 11]. It has been shown that toxic metals induce dysregulation of transcription factors that change site-specific DNA methylation patterns via alteration of DNA accessibility to DNA methylation machinery [12]. Several recent epigenome-wide association studies revealed methylation changes in genes of cellular response to DNA damage (Mn, Cs and Cu) and stress stimulus (Cu and Se), regulation of NF-kappa B signaling (Cs and As), immune and inflammatory responses (Se, Cd and As), cell death and proliferation (Cr, Pb, Mn and As), and estrogen signaling pathway (As) in response to the exposure to metals and metalloids [13–15]. These data are supported by the investigations of blood transcriptome response to environmental heavy metal (Cd, Pb and Hg) exposure which revealed expression changes of multiple genes related to cancer (*RAC1, MAPK1, TP53, UBA52 *and* NFKB1*) and inflammation (*TYK2, JAK3, IGF2, APOA5 *and* STAT3*) [16, 17]. These results are in concordance with the findings of in vitro studies regarding heavy metal-induced gene expression profile changes reported by us and others [18–20]. Using publicly available data, we previously showed altered expression of genes involved in cell proliferation, migration and cell–cell signaling, as well as EGFR signaling, cell cycle control and positive regulation of T- and B-cell proliferation in liver cells in response to in vitro exposure to Cd, Ni and As [20].

Historically, Kapan has been a major center for the production of many non-ferrous metals, and a mining plant has been operating there since 1846 [21]. First publications on heavy metal environmental pollution in this region are dated to 2009 followed by several studies identified that trace elements enter the local food chain in the Kapan mining area (Armenia) [21–28]. Mines, processing plants, active and abandoned tailing repositories and mine water, which local people use for irrigation without any treatment, are significant sources of harmful metals in the mentioned area [22–27]. Particularly, the agricultural soils contain maximum allowed concentration exceeding the contents of Cu, Mo, Ni, Cr, Hg, As and Cd [22–24, 26]. On the territory of the Kapan city, substantial excesses of toxic metal elements of both I (As, Cd, Ni, Pb and Cr) and II–III (Cu, Zn and Mo) categories of hazard were detected [24]. Agricultural crop pollution by Cr, Ni and Pb was also reported in this area previously [24]. Mean concentrations for Hg and Pb in some vegetables and fruits exceed the maximum acceptable levels set by international organizations [24, 27]. The evaluation of combined estimated daily intakes for the local vegetables and fruits revealed exceeded reference doses for Cu and Mo. Moreover, carcinogenic risk values for Ni, Cr, As and Cd exceeded the US Environmental Protection Agency set limits [28]. However, no studies have been performed to determine the blood concentrations of essential and non-essential elements from this mining region residents, and whether there is an epigenetic dysregulation caused by long-term metal exposure.

Based on the previous findings [13–20], genes related to immune response (*APOA5, IGF2, H19 *and* ESR1*) and carcinogenesis (*NFKB1 *and* CDKN2A*) were selected to study possible DNA methylation changes caused by environmental long-term exposure to toxic metals in individuals living in the mining regions of Armenia.

## Results

### Chemical elements analysis in plasma

The blood plasma samples were analyzed by inductively coupled plasma mass spectrometry (ICP-MS) to obtain concentrations of the 29 essential and toxic chemical elements. No detectable levels were found for Bi, Au and Pt in analyzed plasma samples. The measured levels of Cd, Cr, Hg, Sn and Ti were below the limit of quantification (LOQ), and these elements were excluded from the further analyzes. The number of samples < LOQ for four metals were more than half of the total subjects recruited in this study for the following elements: Al (unexposed/exposed; 29/28), Ba (unexposed/exposed; 38/39), Li (unexposed/exposed; 32/33) and Ag (unexposed/exposed; 37/38). These four metals were included in the regression model as categorical variables (< LOQ—absent in plasma and  ≥ LOQ—present in plasma).

**Fig. 1:**
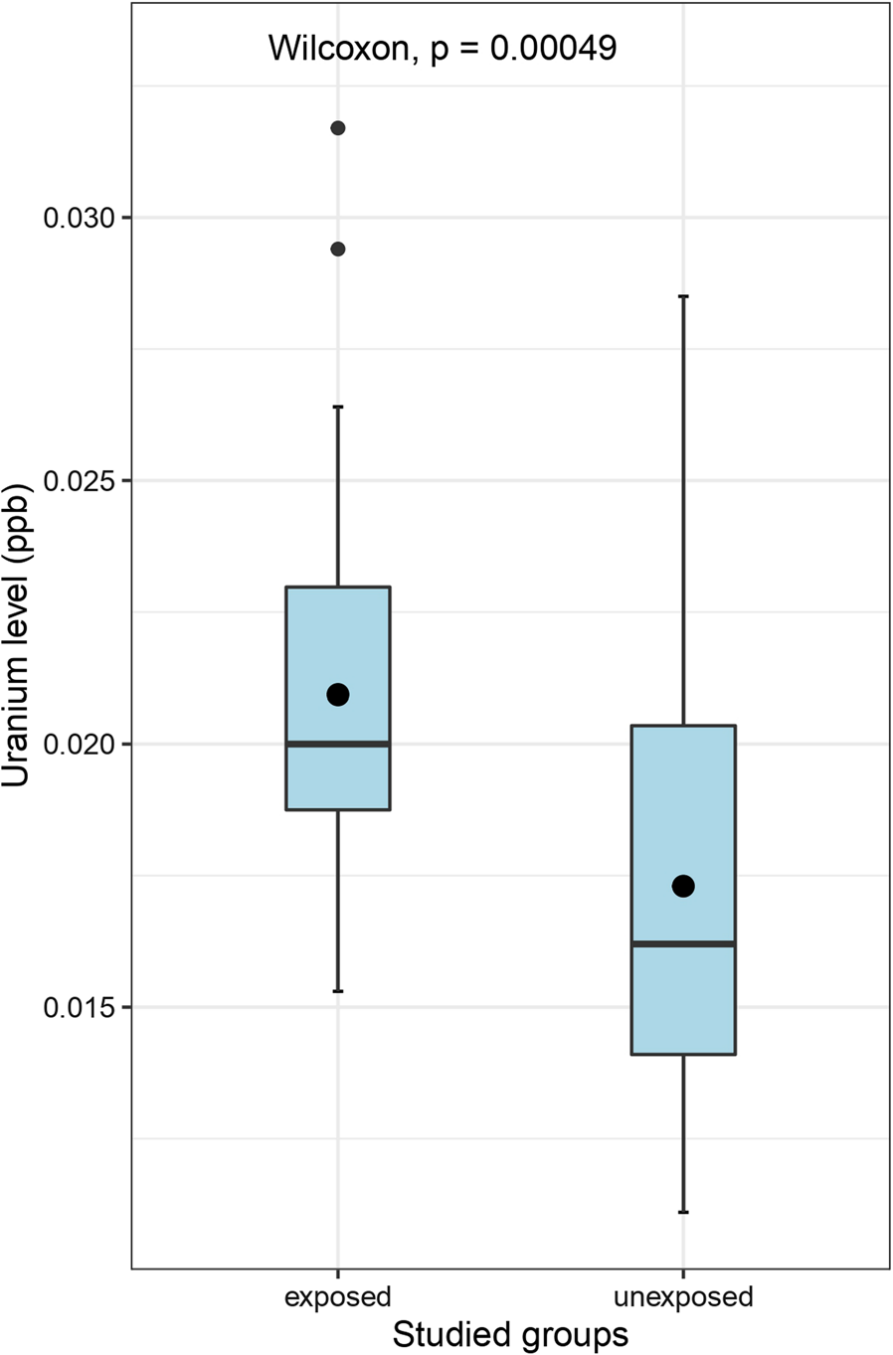
Difference of uranium plasma levels between exposed and unexposed subjects

There was a significantly higher plasma *U* level in the exposed group compared to unexposed (*p* = 0.00049, Fig. 1). No statistically significant differences were found by non-parametric testing (Mann–Whitney *U*) between the two studied groups for the remaining 16 elements (Table 1).

**Table 1:** Clinical and demographic characteristics of the study groups

Characteristics	Exposed individuals^a^ (*n* = 40)	Unexposed individuals^b^ (*n* = 40)	*p* value
	Number (%) or median (IQR)	Number (%) or median (IQR)	
Age (years)	35.5 (32, 38.25)	32 (31, 36)	0.01
Gender			
Female	25 (62.5%)	22 (55%)	0.53
Smoking			
Smokers	13 (32.5%)	14 (35%)	0.88
BMI (kg/m^2^)	27.2 (22.87, 29.76)	24.25 (21.16, 28.67)	0.06
BMI class			0.05
Normal (18.5–25 kg/m^2^)	15 (37.5%)	24 (60%)	
Overweight (25–30 kg/m^2^)	17 (42.5%)	12 (30%)	
Obesity (≥ 30 kg/m^2^)	8 (20%)	4 (10%)	
Chemical elements in plasma (ppb)			
Ca	112 × 10^3^(106 × 10^3^,116 × 10^3^)	110 × 10^3^(103 × 10^3^, 117 × 10^3^)	0.37
Mg	23 × 10^3^ (22 × 10^3^, 24 × 10^3^)	23 × 10^3^ (21 × 10^3^, 24 × 10^3^)	0.18
Co	0.41 (0.30, 0.60)	0.49 (0.37, 0.63)	0.09
Fe	2165 (1655, 2815)	2280 (1760, 2690)	0.95
I	81 (74, 86)	75 (69, 84)	0.06
Mn	2.7 (2.4, 3.2)	2.6 (2.4, 3.3)	0.78
Cu	1240 (1100, 1340)	1170 (1010, 1280)	0.40
Mo	1.15 (0.90, 1.90)	1.24 (1.03, 1.74)	0.26
Se	128.5 (118, 141)	135 (126, 142)	0.18
Cr	< 8.312 (LOQ)		NA
Zn	1540 (1410, 1780)	1590 (1440, 1670)	0.97
Cd	< 0.378 (LOQ)		NA
As	2 (2, 2)	2 (2, 3)	0.23
Hg	< 4.042 (LOQ)		NA
Sb	8 (7, 9)	8 (7, 9)	0.73
Tl	0.026 (0.015, 0.035)	0.031 (0.021, 0.045)	0.07
Al	260 (240, 315)	260 (230, 310)	0.31
Ba	2 (2, 2)	2 (2, 3)	0.42
Be	0.2 (0.1, 0.2)	0.2 (0.1, 0.2)	0.48
V	4 (4, 4)	4 (4, 4)	0.27
Bi	< 0.002 (LOD)		NA
Au	< 0.06 (LOD)		NA
Li	4 (4, 5)	4 (3, 5)	0.09
Ni	9 (8, 9)	9 (8, 10)	0.52
Sn	< 2.558 (LOQ)		NA
Pt	< 0.003 (LOD)		NA
Ag	0.14 (0.10, 0.10)	0.19 (0.10, 0.10)	0.86
Ti	< 12.259 (LOQ)		NA
U	0.020 (0.018, 0.023)	0.016 (0.014, 0.021)	0.00049

*LOD* limit of detection, *LOQ* limit of quantification, *IQR* interquartile range and *NA* not applicable

^a^Exposed individuals are from the mining areas: Syunik village, Artsvanik village and Kapan city (Armenia)

^b^Unexposed individuals are recruited from the Yerevan city (Armenia)

Inter-elemental correlation analysis revealed clusters of elements correlated with each other in both, exposed and unexposed groups (Additional file 1: Figures S2–S3). The following clusters of a moderate positive relationship between elements were found for the exposed group: (Mn, Fe, Zn and Sb); (Mn, Al and Ba); (Cd, Se and Sn) and (As, Sn and Tl). The correlation matrix of the unexposed group contains two positively correlated clusters of (Mn, Fe and Zn) and (Al, Ni and Sn) (correlation coefficient ≥ 0.4) (Additional file 1: Figures S2–S3).

### Differentially methylated positions

**Fig. 2:**
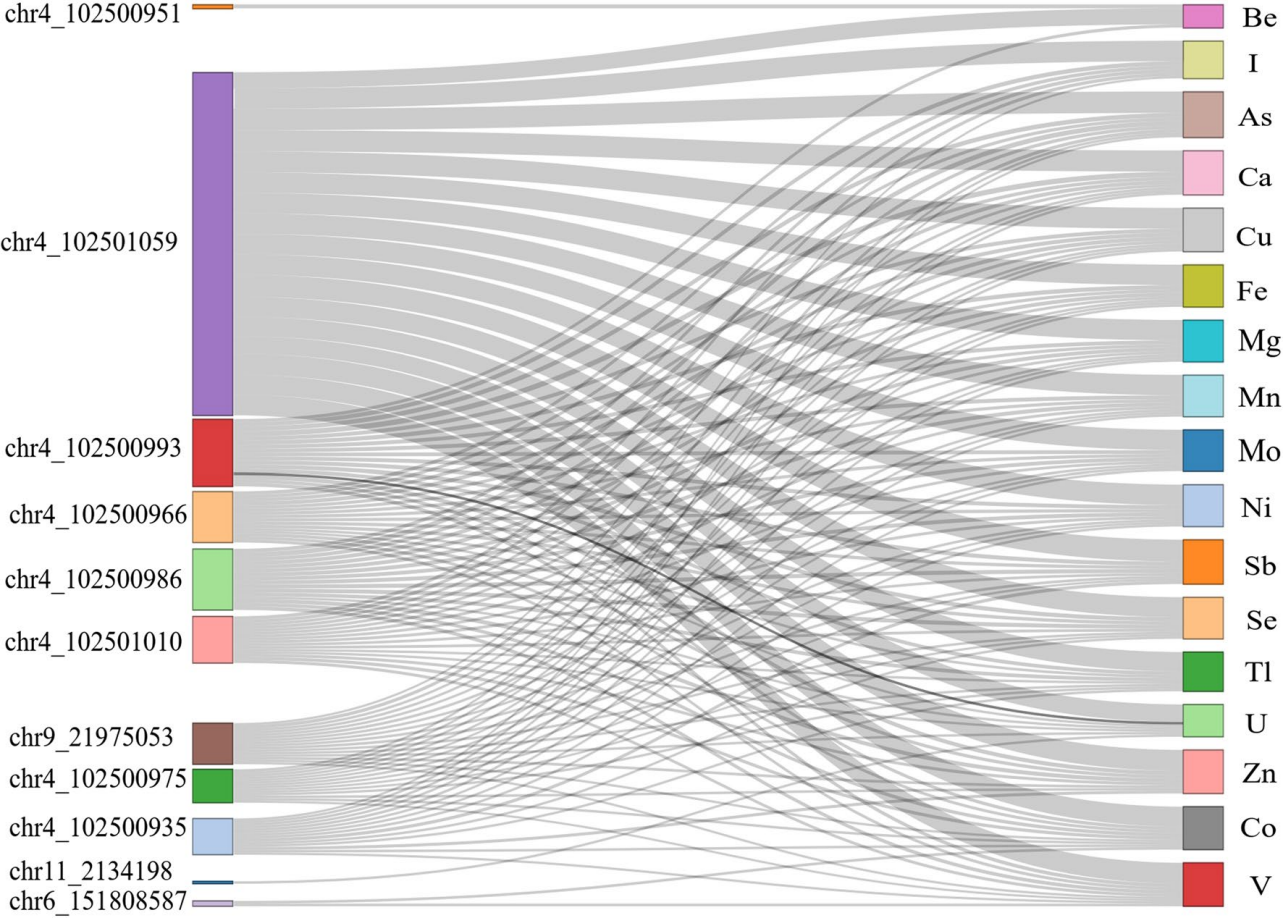
Differentially methylated positions in association with the plasma levels of chemical elements. Sankey diagram visualizes the association between methylation status of deferentially methylated CpGs and plasma concentrations of chemical elements. The x-axis represents differentially methylated CpGs and chemical elements. The nodes on y-axis represent the positions of CpGs deferentially methylated in association with chemical elements, number of later is proportional to the size of each node and width of each arc

Overall, the methylation level of the 654 CpG dinucleotides located on *NFKB1, CDKN2A, ESR1, APOA5, IGF2 *and* H19* genes was measured in this study for all samples from exposed and unexposed groups (Additional file 2: Spreadsheet 3). In the linear model adjusted for participants’ gender, age, BMI, BMI class and smoking, heavy metal exposure was associated with hypomethylation of five CpG positions in the *NFKB1* gene (CpGs at 102501059, 102500993, 102501010, 102500986 and 102500966 positions on chromosome 4) (Additional file 2: Spreadsheet 3). Moreover, these associations remained significant after adding 20 metal levels as cofactors in this model. The methylation levels of these core five CpGs in *NFKB1* gene showed negative correlation with the levels of uranium in plasma (Additional file 1: Figure S4). In case of Be exposure-related regression, coefficients for CpGs at 102501010, 102500986 and 102500966 were non-significant (Fig. 2, Additional file 2: Spreadsheet 4). Additional CpGs were found to be differentially methylated in a regression model upon the inclusion of metal plasma levels. Thus, chr9:21975053 at *CDKN2A* gene was differentially methylated in exposed group vs unexposed group upon inclusion of multiple metals (Ba, I, Al, As, Ca, Ag, Cu, Fe, Li, Mg, Mn, Mo, U, Ni, Sb, Se, Tl, Zn, Co and V); *NFKB1* gene (chr4:102500935 and chr4:102500975) was differentially methylated upon inclusion of Al, As, Ca, Ag, Cu, Fe, Li, Mg, Mn, Mo, U, Ni, Sb, Se, Tl, Zn, Co and V (Fig. 2, Additional file 2: Spreadsheet 4). The CpG at chr6:151808587 position of *ESR1* gene was significantly hypomethylated when adjusted with Co and V plasma levels (Fig. 2, Additional file 2: Spreadsheet 4). As for DMPs associated with specific metals, *NFKB1* gene chr4:102500951 and *IGF2* gene chr11:2134198 site CpGs were hypermethylated when adjusted to Be and U plasma levels, respectively (Fig. 2, Additional file 2: Spreadsheet 4). No association of methylation status of *APOA5* gene CpG islands with heavy metal exposure was found in our study (Fig. 2, Additional file 2: Spreadsheet 4).

### Methylations of CpGs associated with plasma levels of chemical elements

**Fig. 3:**
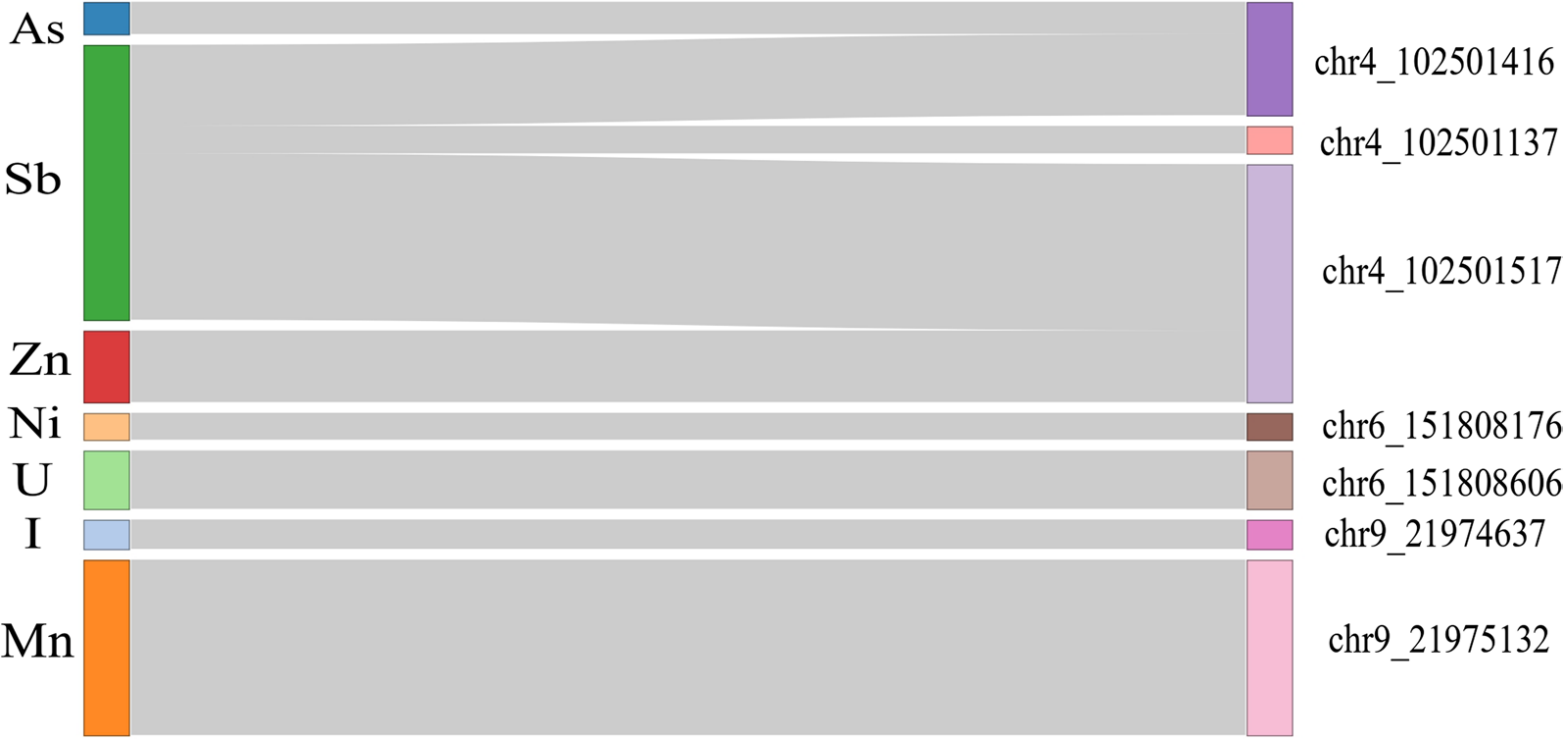
CpGs associated with plasma levels of chemical elements. Sankey diagram visualizes the association between methylation status of CpGs (right) and plasma concentrations of chemical elements (left)

Overall, the methylation levels of the seven CpG sites were associated with plasma levels of seven chemical elements in all participants involved in this study (Fig. 3, Additional file 2: Spreadsheet 5). Three CpGs at *NFKB1* gene: chr4:102501416, chr4:102501137 and chr4:102501517 sites were associated with As, Sb and Zn plasma levels, respectively. Methylation levels of the two CpG dinucleotides at *ESR1* gene: chr6:151808176 and chr6:151808606 showed positive association with Ni and U plasma levels, respectively. Finally, *CDKN2A* gene chr9:21974637 and chr9:21975132 cytosines were hypermethylated when associated with the I and Mn levels in plasma, respectively (Fig. 3, Additional file 2: Spreadsheet 5).

## Discussion

This study explored the effects of long-term human environmental exposure to heavy metals on DNA methylation of immune response and carcinogenesis-related genes. We identified five positions of CpGs (core CpGs) in the *NFKB1* promoter region which were hypomethylated in the exposed group compared to unexposed. Overall, we found four DMPs associated with multiple metals as well as two DMPs with specific metal exposure. Accordingly, two of them are located on the *NFKB1* gene (all metals, except Be, Ba and I), one on the *CDKN2A* gene (all metals, except Be) and one on the *ESR1* gene (Co and V). The DMPs associated with specific metal levels are located on *NFKB1* (chr4:102500951, associated with Be) and *IGF2* (chr11:2134198, associated with U). Additionally, we identified CpGs at which DNA methylation was associated with metal plasma levels for all groups involved in this study. In general, the methylation status of the seven CpGs located on *NFKB1* (3)*, ESR1* (2)and* CDKN2A* (2) was positively correlated with plasma levels of seven metals (As, Sb, Zn, Ni, U, I and Mn).

The core CpGs, hypomethylated in the exposed group, were located on the promoter of *NFKB1* gene encoding 105-kD transcription factor (TF) precursor, which undergoes cotranslational processing to form DNA-binding p50 protein [29]. The p50 protein acts in NF-κB signaling pathway as a transcription activator or repressor, assembling with Rel-proteins or by homodimerization, respectively [29]. NF-κB plays a crucial role in cell survival, growth, immune response and inflammation since it controls cell response to stress, free radicals, irradiation and foreign antigens [30, 31]. Several previous epigenome-wide association studies (EWAS) reported the involvement of NF-κB pathway genes in DNA methylation changes induced by heavy metal environmental, occupational and prenatal exposure [13, 14, 32]. Also, a recent study on arsenic-exposed 396 Bangladeshi adults revealed among differentially methylated genes significant enrichment of those annotated to reactive oxygen species pathway, inflammatory response and nuclear factor kappa-B signaling [14]. Zeng et al. reported differential methylation of 125 CpGs mapped to 79 genes in the e-waste-exposed group with higher concentrations of Pb, Cd, Mn and Cr in neonatal umbilical cord blood [32]. These genes are involved in signaling pathways related to NF-κB activation, adherens junction, TGF beta and apoptosis [33]. Another study showed that prenatal exposure to 12 metals (As, Cd, Cr, Cs, Cu, Hg, Mg, Mn, Se and Zn) associated with DMPs located in genes associated with the regulation of NF-κB signaling as well as several biological processes such are neurodevelopment, inflammatory response, cellular response to stress and DNA damage and apoptosis [13]. In addition to the CpGs differentially methylated in response to specific metals, the authors reported DMPs annotated to genes in human leukocyte antigen (HLA) region overlapping for Cr, Cs, Cu, Hg, Mg and Mn metals [13]. Importantly, the toxicological impact of metals is relevant to mixtures and concentrations they co-exist in the environment [33]. The study found that in T-cell culture, arsenic exposure only vs exposure to arsenic and uranium mixture activates different signaling pathways, including NF-κB activation in response to arsenic and uranium co-exposure [33]. In our study, we found two DMPs located in the *NFKB1* promoter region, which were associated with multiple metals exposure, and one CpG dinucleotide which was differentially hypomethylated in response to Be. Though no study to date has reported epigenetic dysregulation related to Be environmental exposure, beryllium fluoride in vitro on murine peritoneal macrophages at low concentration affects the level of NF-κB and by elevating Ca^2+^ triggers the activation of p21^ras^-dependent MAPK signaling cascade [34].

Our work is the first study providing the reference values of a wide range of essential and non-essential trace elements in the blood plasma of adults from the Armenian population. Compared to other data provided by the studies done to date, generally, the obtained concentrations were comparable to other countries. However, there were trends of increased plasma levels for several non-essential trace elements, such as nickel, antimony and vanadium. Thus, relatively high Ni and Sb levels were detected in our study (9 and 8 ppb, respectively) compared to those reported for populations in Germany (0.11 and < 0.013 ppb, respectively), Australia (2 and 4 ppb, respectively) and France (1.49 and 0.25 ppb, respectively) [35–37]. Plasma concentrations of V found for Armenians (4 ppb) were higher than in Germans and Australians described earlier (0.052 and 0.2 ppb, respectively) [35, 36]. Obtained population differences of trace element plasma concentrations might be associated with genetic as well as environmental, geographical, nutritional and lifestyle features of the Armenian population [38, 39].

According to the correlation plots, different clusters of moderate positively correlated metals were found in the metal-exposed versus unexposed group. The inter-elemental correlations for exposed subjects have greater strength and higher number of clusters compared to unexposed ones. The obtained dissimilarity of the correlation matrices between the two study groups might reflect the different local environments of the people from the mining region and unexposed subjects.

Elevated levels of uranium in the exposed group found in our study were associated with methylation changes of CpG sites in *NFKB1*, *IGF2* and *CDKN2A* genes. Very few studies to date have been carried out to investigate the influence of uranium exposure on epigenetic changes in the human genome. Uranium-induced aberrant global DNA methylation was reported by different in vivo studies as well as by investigations on epigenetic dysregulations caused by depleted uranium human low-dose exposure [40–42]. The inactivation of *CDKN2A* tumor suppressor gene and *MGMT* (O6-methylguanine-DNA methyltransferase) repair gene by altered promoter methylation among Chinese miners of U has been recently found [43]. These results are in concordance with our finding on hypomethylation of intronic CpG of *CDKN2A* gene. *CDKN2A* gene locus encodes two tumor suppressor proteins p16/INK4A and P14/ARF, which regulate cell cycle by cyclin D inhibition, and its inactivation leads to a tumorigenesis through the PI3K/Akt pathway [44]. Recent studies provide evidence of PI3K and MAPK pathways' involvement in uranium-induced toxicity [45, 46]. In addition, the activation of the NF-κB/IL-6 pathway in response to INK4a/ARF inhibition triggering tumor growth and development has been described recently [47]. Further, co-exposure of sodium arsenite and uranyl acetate alters gene expression in T cells by positive regulation of NF-κB transcription factors [33]. The oxidative stress-induced activation of the NF-κB leads to *IGF2* hypermethylation in mice models [48]. A highly significant increase in the expression of the *IGF2* gene in response to exposure to five heavy metals (Cd, Cr, Cu, Pb and Zn) was reported in fishermen's blood [49]. The *IGF2* gene epigenetic dysregulation caused by heavy metals exposure was found by different groups in the previous studies as well [50, 51]. In the current study, we revealed that U and Ni levels were independently correlated with hypermethylation of CpGs located in the intron2 and exon3 of the *ESR1* gene, respectively. Further, we found the intronic DMP in the *ESR1* gene to be associated with Co and V plasma levels and hypomethylated in the exposed group. Evidence obtained from epidemiological, in vivo and in vitro studies suggests activation of estrogen receptor signaling pathway as well as alterations of *ESR1* gene methylation status in response to heavy metals exposure [14, 52, 53]. According to the recent EWAS on arsenic environmental exposure, estrogen early response was among pathways of the annotated genes with arsenic-associated CpGs [14]. In addition, the evidence of overexpression of *ESR1* gene resulting from arsenic-induced promoter region hypermethylation was observed in activated hepatocellular proliferation [53]. Further, it was shown that Cd treatment stimulates breast cancer cell proliferation by activating ERα-dependent PI3K-Akt signaling pathway [52]. Drinking water with a low dose of uranium causes estrogen receptor (ER)-dependent responses in female mice [52]. One possible explanation of the growing evidence of heavy metal-induced ER signaling activation might be the ability of some metals, so-called metalloestrogens, including Ni, Co and V, to mimic estrogen [54]. By binding to ERα, these metals activate it and lead to both direct transcription activation of target genes in the nucleus or switch rapid nongenomic pathways via signaling of mitogen-activated protein kinases (MAPKs) [55].

Altogether, with supports of our study results on epigenetic changes at *NFKB1, IGF2, ESR1* and *CDKN2A* genes, these findings elucidate the dysregulation of PI3K, MAPK and NF-κB pathways in alterations of immune response and oncogenesis induced by heavy metal exposure [56]. However, further research is needed to confirm these results and to find out the epigenetic mechanisms of the heavy metal-induced alterations of aforementioned signaling pathways.

No association of methylation status of *APOA5* gene CpG islands with heavy metal exposure was found in our study. However, it was previously reported about significant non-promoter-associated increases in DNA methylation of *APOA5* in blood cells of Pb-exposed women’s grandchildren [57]. The absence of association found in our study might be partially explained by hypermethylation and lack of mRNA of *APOA5* in blood cells [58]. Another reason might be that Pb blood level was not investigated in our study.

This study has several limitations. The first limitation is the moderate sample size, based on the strict selection of subjects living long-term in the mining area. In addition to metals investigated in this study, other metals and possible toxic agents/pollutants and their combinations may induce epigenetic changes and should be investigated in the future studies.

## Conclusions

In the current study, we investigated the effect of long-term heavy metal exposure on methylation changes in human leukocytes. We identified hypomethylation of five CpG sites in the *NFKB1* promoter region in the mining region residents group. Additionally, methylation changes of CpG sites in *NFKB1, CDKN2A, IGF2* and *ESR1* genes to be associated with multiple and specific metal exposure. The consequences of these heavy metals long-term exposure on gene expression and NF-κB pathway should be further investigated. New studies must be carried out on larger sample cohorts to replicate our results.

## Methods

### Study population

Exposed and unexposed subjects (*n* = 80) were selected based on the distance to the mine as well as reports on heavy metal levels in the habitation area [23, 24]. In total, 40 subjects (M/F; 15/25, age (mean, min–max); 35.6, 29–43), who are living near the mining region and regularly consume vegetables, fruits and dairy with trace elements concentrations exceeding the maximum acceptable levels (11—Syunik village, 11—Artsvanik village and 18—Kapan city, Armenia) were recruited in a metal-exposed group (Additional file 1: Figure S1). In this area, the reported environmental high level of heavy metals is caused by anthropogenic pressure as well as natural geogenic source [22–26, 59]. Forty subjects (M/F; 18/22, age (mean, min–max); 33.4, 28–42) living in Yerevan city, Armenia, were recruited in the unexposed group since there is no mining activity around the city. All subjects were of Armenian descent and had lived their entire lives in the respective location (Table 1).

The subjects were selected based on a questionnaire that included information about age, residence time in the respective locality, smoking habit, use of drugs, alcohol, illnesses and chest X-ray. The following exclusion criteria were used: (a) metabolic and acute inflammatory diseases; (b) the use of mineral supplements; (c) excessive alcohol consumption; (d) the presence of metal implants; (e) vegetarianism and other specific eating habits; (f) surgery or exposure to radiation or any chemicals at least a month before sampling and (g) pregnancy. BMI was calculated using the standard formula: The body mass divided by the square of the body height. BMI categories were defined according to the standards of the World Health Organization (WHO) [60].

The details of demographic and clinical characteristics of the study groups are presented in Table 1.

At the time of sample collection, all subjects provided informed consent about the use of the blood for planned analysis. The study was approved by the Ethic Committee (IRB/IEC:IRB00004079, Approval#1/2020).

### Analysis of the chemical elements in plasma

Plasma samples of 80 subjects recruited in this study were separated from whole blood by centrifugation (2000×*g*, 10 min, 4 °C) and stored at − 40 °C for further chemical analysis.

Preprocessing of the plasma samples was performed before analysis by adding 15 volumes of diluent (1% 1-butanol, 0.1% Triton × 100 and 0.07% HNO_3_ in distilled deionized water (18 MΩ·cm) pH = 2.0) to the 0.8 mL of plasma as described earlier [61].

The concentrations of 29 essential and toxic/potentially toxic trace elements (calcium (Ca), magnesium (Mg), cobalt (Co), iron (Fe), iodine (I), manganese (Mn), copper (Cu), molybdenum (Mo), selenium (Se), chromium (Cr), zinc (Zn), cadmium (Cd), arsenic (As), mercury (Hg), antimony (Sb), thallium (Tl), aluminum (Al), barium (Ba), beryllium (Be), vanadium (V), bismuth (Bi), gold (Au), lithium (Li), nickel (Ni), tin (Sn), platinum (Pt), silver (Ag), titanium (Ti) and uranium (U)) in plasma samples were analyzed with NexION 300D (PerkinElmer Inc., Shelton, CT 06484, USA) ICP-MS. The Dynamic Reaction Cell (DRC) technology was used for the removal of atomic interferences. Calibration of the system was done according to the manufacturer’s guidelines using Standards Kits (PerkinElmer Inc., Shelton, CT 06484, USA). The online internal standardization with rhodium-103 was performed to account for the incomplete acidity and viscosity matching between calibration and sample matrices. Intralaboratory control was performed using the reference materials ClinCheck Plasma Control (RECIPE Chemicals + Instruments GmbH, Germany).

We applied the following quality control measures: A coefficient of variation (CV) was below 15% for intra- and inter-day precision, the recovery rates for all elements were within the limit of 90–110%. The limit of detection (LOD) and LOQ values for each element were measured (Additional file 1: Table S1). In this analysis, we include only elements that have ≥ LOQ.

### DNA extraction and bisulfite treatment

Peripheral venous blood samples were collected in K3EDTA tubes (4 mL) from the metal-exposed and unexposed individuals. Genomic DNA was isolated from peripheral blood cells according to the standard salting out method with a modification of adding chloroform step [62]. All DNA samples had A260/A280 ratio within 1.8–2.0 range and a concentration of more than 50 ng/μL.

The EZ DNA Methylation-Gold™ Kit (Zymo Research, USA) was used for the bisulfite conversion of DNA samples. Bisulfite treatment of samples was done according to the manufacturer’s instructions with some changes at incubation and elution stages to reduce salts in final bisulfite-converted DNA samples (BS-DNA) and degradation of DNA. Thus, the first incubation was done as 90 °C for 10 min, and elution with 20 μL of TE buffer was followed by centrifugation 5 min at 2500*g* at the last step. The input of DNA was between 400 and 500 ng, and quantification of BS-DNA was done by spectrophotometer with RNA settings.

To control the success of the conversion and possible fragmentation of BS-DNA, the BS-PCR was performed with primers targeting different lengths of template DNA (Additional file 1: Table S2). The product sizes were analyzed by the microchip electrophoresis system (MCE™-202 MultiNA, Shimadzu, Japan).

### Library preparation and sequencing

The regions of interest in the *ESR1, IGF2, H19, APOA5, NFKB1* and *CDKN2A* genes were selected based on sequences from UCSC genome browser (Genome Reference Consortium Human Build 38) [63] and literature review. By design, targets were enriched for differentially methylated regulatory regions and TF binding sites, which predominantly carry H3K27ac and H3K4me3 annotations, mainly located in promoters, CpG Islands (Additional file 2: Spreadsheet 1). The primer design for target locations was performed using BS-DNA as a template with the online tool BiSearch [64] and the following restrictions: (a) length of PCR product up to 290 bp; (b) the similar Tm for primer pairs and among all primers, preferably ± 2 °C and (c) length of primers: from 15 up to 35 bases.

To evaluate the designed primers, the BS-PCR was performed with genomic DNA and water as a negative control as well as two quality control primers. The product sizes were analyzed by the MultiNA microchip electrophoresis system to exclude non-specific amplification. Further, 54 pairs of bisulfite-specific primers (Additional file 1: Table S2) were selected and used for target enrichment applying to the 48.48 Access Array Integrated Fluidic Circuit (Fluidigm, USA). In total, 54 indexed amplicons were synthesized for each sample and deep-sequenced on MiSeq (Illumina, USA) as reported previously [65].

### Data processing

BiSulfite Bolt (BSBolt) was used for the alignment of sequencing reads to a reference of hg38 genome [66]. To distinguish true C/T from unmethylated cytosines, BS-SNPer was used for more accurate quantification of methylation levels [67]. Methylation levels in CpG and CHH (H = A, C or T) context with a minimum quality score of 20 were calculated and subjected to the following quality control steps: (a) The samples with more than 98% conversion efficiency considering methylation levels at CHH context were included in the analysis (Additional file 2: Spreadsheet 2); (b) PCR artifacts and non-specific PCR products were excluded from analysis and (c) coverage cutoff of 1000 reads, CpGs with less than 1000 reads were excluded from downstream analysis.

### Statistical analysis

Medians and interquartile ranges (IQRs) for continuous variables and frequencies/proportions for categorical variables were calculated for the clinical and demographic characteristics of the study groups. The differences between metal-exposed and unexposed groups were assessed by applying the Mann–Whitney test for continuous variables and the Chi-squared test for categorical variables. Spearman correlation was used to analyze relationships between concentrations of metals in exposed and control groups. The M-value was calculated for each CpG position as the log2 ratio of the reads containing methylated versus unmethylated cytosine for a given CpG position [68]. Linear regression was conducted to find DMPs using the R package limma [69] with adjustment for gender, age, BMI class, smoking and chemical elements concentration (for each element separately). The Benjamini–Hochberg false discovery rate (FDR) method for adjustment of p values for multiple comparisons (FDR < 0.05 is retained as significant) was used.

### Supplementary Information

**Additional file 1**: This file contains information about the detection limits of chemical elements measured in plasma (Table S1), bisulfite-specific primers and target regions spanning CpG islands (Table S2), map of the polluted region where exposed subjects are living (Figure S1), correlation matrices of chemical elements for exposed group and unexposed groups (Figures S2 and S3, respectively), correlation matrix of metal plasma levels and methylation levels of CpGs associated with metal exposure (Figure S4) and reference levels of 26 chemical elements in human blood plasma for both genders in Armenian population (Table S3).

**Additional file 2**: Additional spreadsheets present information about positions and regions of DMPs and conversion rate and raw read numbers for each sample included in the current study (Additional spreadsheets 1 and 2, respectively). Further, this file contains the results of differential methylation analysis, DMPs associated with chemical elements blood levels as well as CpGs associated with elements concentration obtained by linear regression analysis adjusted for gender, age, BMI class, smoking and each element separately (Additional spreadsheets 3, 4 and 5, respectively).

## Data Availability

The raw sequencing data obtained by our study are in process of submission to dbGaP.

## Abbreviations

Ag: Silver
Al: Aluminum
As: Arsenic
Au: Gold
Ba: Barium
Be: Beryllium
Bi: Bismuth
BS-DNA: Bisulfite-converted DNA samples
Ca: Calcium
Cd: Cadmium
Co: Cobalt
Cr: Chromium
Cu: Copper
CV: Coefficient of variation
DMP: Differentially methylated position
ER: Estrogen receptor
EWAS: Epigenome-wide association studies
FDR: False discovery rate
Fe: Iron
Hg: Mercury
HLA: Human leukocyte antigen
I: Iodine
ICP-MS: Inductively coupled plasma mass spectrometry
IQR: Interquartile range
Li: Lithium
LOD: Limit of detection
LOQ: Limit of quantification
MAPK: Mitogen-activated protein kinase
Mg: Magnesium
Mn: Manganese
Mo: Molybdenum
Ni: Nickel
Pt: Platinum
Sb: Antimony
Se: Selenium
Sn: Tin
TF: Transcription factor
Ti: Titanium
Tl: Thallium
U: Uranium
V: Vanadium
WHO: World Health Organization
Zn: Zinc
