# Correction: Long-term environmental metal exposure is associated with hypomethylation of CpG sites in *NFKB1* and other genes related to oncogenesis

**DOI:** 10.1186/s13148-023-01559-w

**Published:** 2023-09-05

**Authors:** Ani Stepanyan, Anna Petrackova, Siras Hakobyan, Jakub Savara, Suren Davitavyan, Eva Kriegova, Arsen Arakelyan

**Affiliations:** 1 Institute of Molecular Biology, National Academy of Sciences, Yerevan, Republic of Armenia; 2 Department of Immunology, Faculty of Medicine and Dentistry, Palacký University Olomouc and University Hospital Olomouc, Olomouc, Czech Republic; 3 Department of Computer Science, Faculty of Electrical Engineering and Computer Science, VSB-Technical University of Ostrava, Ostrava, Czech Republic

**Correction to: Clinical Epigenetics (2023) 15:126** 10.1186/s13148-023-01536-3

Following publication of the original article [1], the author noticed that the descriptions of Figs. 2 and 3 are placed incorrectly in the textual part instead of figure captions.

**Fig. 2:**
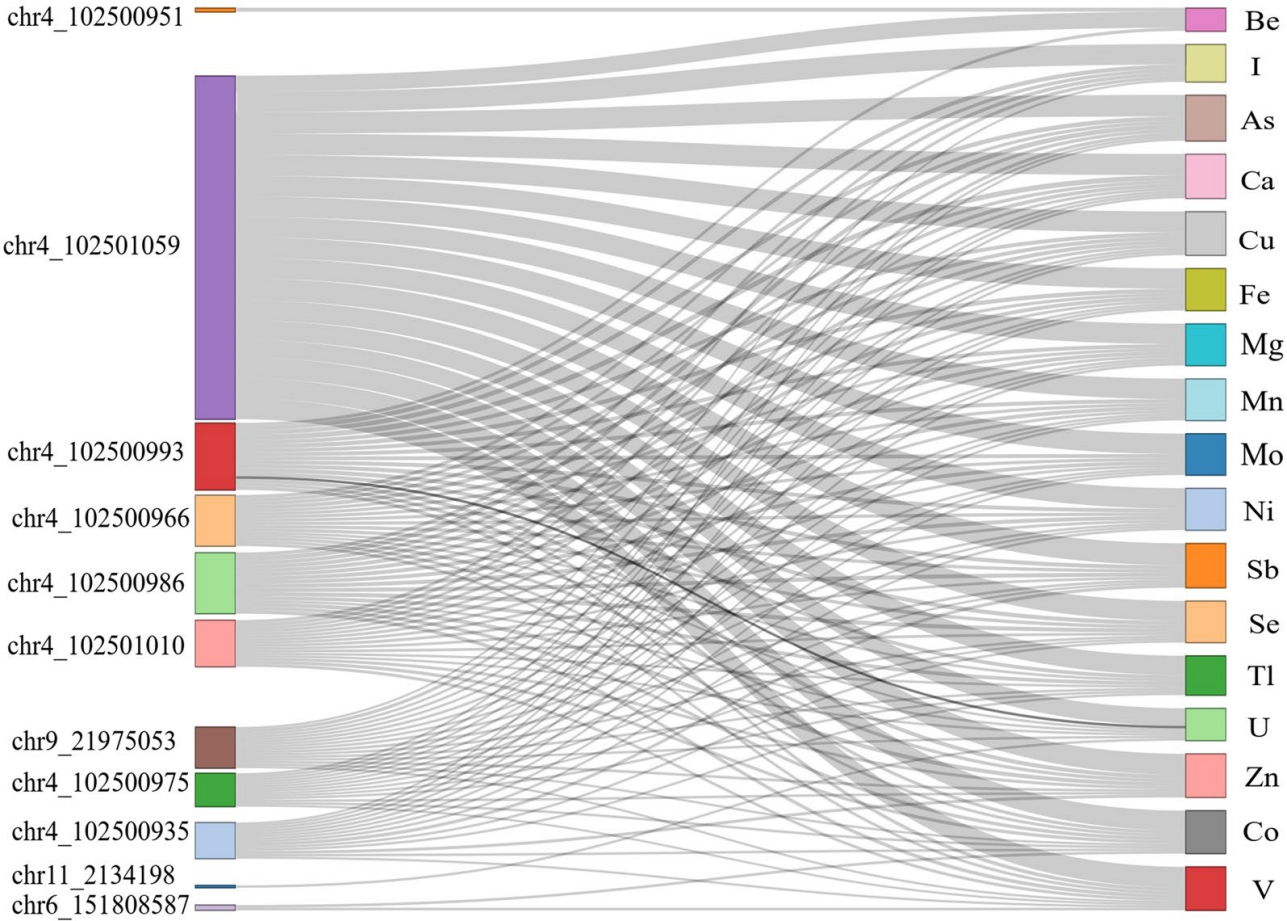
Differentially methylated positions in association with the plasma levels of chemical elements. Sankey diagram visualizes the association between methylation status of deferentially methylated CpGs and plasma concentrations of chemical elements. The x-axis represents differentially methylated CpGs and chemical elements. The nodes on y-axis represent the positions of CpGs deferentially methylated in association with chemical elements, number of later is proportional to the size of each node and width of each arc

**Fig. 3:**
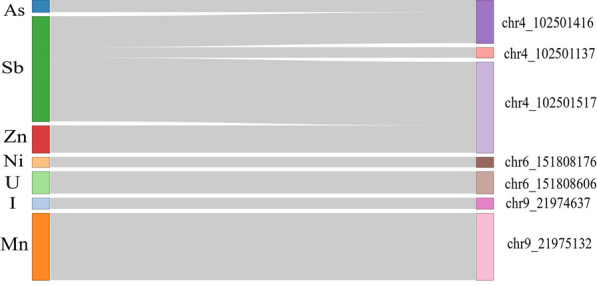
CpGs associated with plasma levels of chemical elements. Sankey diagram visualizes the association between methylation status of CpGs (right) and plasma concentrations of chemical elements (left)

This has now been corrected with this erratum.

The original article has been corrected.

## References

[1] Stepanyan A, Petrackova A, Hakobyan S, Savara J, Davitavyan S, Kriegova E, Arakelyan A (2023). Long-term environmental metal exposure is associated with hypomethylation of CpG sites in *NFKB1* and other genes related to oncogenesis. Clin Epigenet.

