# Supplementary material for: Long-term environmental metal exposure is associated with hypomethylation of CpG sites in NFKB1 and other genes related to oncogenesis
Source: Clin Epigenetics. 2023 Aug 7;15:126. doi: 10.1186/s13148-023-01536-3 (PMC10405444; doi:10.1186/s13148-023-01536-3)
Supplement: Supplementary file 1 — Additional file 1: This file contains information about the detection limits of chemical elements measured in plasma (Table S1), bisulfite-specific primers and target regions spanning CpG islands (Table S2), map of the polluted region where exposed subjects are living (Figure S1), correlation matrices of chemical elements for exposed group and unexposed groups (Figures S2 and S3, respectively), correlation matrix of metal plasma levels and methylation levels of CpGs associated with metal exposure (Figure S4) and reference levels of 26 chemical elements in human blood plasma for both genders in Armenian population (Table S3). [file 13148_2023_1536_MOESM1_ESM.docx]

**ADDITIONAL MATERIAL**

**Title:** Long-term environmental metal exposure is associated with hypomethylation of CpG sites in *NFKB1* and other genes related to oncogenesis

**Authors:** Ani Stepanyan, Anna Petrackova, Siras Hakobyan, Jakub Savara, Suren Davitavyan, Eva Kriegova, Arsen Arakelyan

**Table S1.** Detection limits of chemical elements measured in plasma

| Element | Mass | Detection limits | |
| --- | --- | --- | --- |
|  |  | LOD ppb | LOQ ppb |
| Li | 7 | 0.034 | 3.424 |
| Be | 9 | 0.001 | 0.132 |
| Mg | 25 | 0.166 | 31.117 |
| Al | 27 | 0.166 | 31.107 |
| Ca | 42 | 0.289 | 54.308 |
| Ti | 50 | 0.065 | 12.259 |
| V | 51 | 0.0007 | 0.136 |
| Cr | 53 | 0.044 | 8.312 |
| Mn | 55 | 0.008 | 1.627 |
| Fe | 57 | 0.182 | 34.050 |
| Co | 59 | 0.001 | 0.241 |
| Ni | 60 | 0.012 | 2.187 |
| Cu | 65 | 0.021 | 3.921 |
| Zn | 67 | 0.597 | 111.959 |
| As | 75 | 0.008 | 1.621 |
| Se | 78 | 0.017 | 3.342 |
| Mo | 98 | 0.003 | 0.599 |
| Ag | 109 | 0.001 | 0.131 |
| Cd | 111 | 0.002 | 0.378 |
| Sn | 120 | 0.013 | 2.558 |
| Sb | 121 | 0.002 | 0.407 |
| I | 127 | 0.238 | 44.726 |
| Ba | 137 | 0.056 | 10.673 |
| Pt | 195 | 0.0006 | 0.118 |
| Au | 197 | 0.001 | 0.246 |
| Hg | 202 | 0.021 | 4.042 |
| Tl | 205 | 0.0004 | 0.007 |
| U | 238 | 0.0005 | 0.013 |

**Table S2**. Bisulfite-specific primers and target regions spanning CpG islands

| gene | CpG island  position / CpG count | primers (5' to 3') | amplicon start/end (hg38) |
| --- | --- | --- | --- |
| *NFKB1* | chr4:102500935-102502072 / 130 | TAGTTAGGAAGTGAGAGAGTGA | chr4:102501319-102501448 |
|  |  | ACR*AAAAAAACAAAAAAAACTAT |  |
|  |  | TTTTAGTTTTTGGGATTY**GG | chr4:102501042-102501333 |
|  |  | CTCACTTCCTAACTAAAAAT |  |
|  |  | GTTTAGTTTTTYGTATTTTTTTTAG | chr4:102501847-102502145 |
|  |  | CACTCCAACCTTCTCACCAT |  |
|  |  | GTTTTATTTTTATATTTGGTATG | chr4:102500857-102501086 |
|  |  | ACTCTAACTTCCTAACAAAA |  |
|  |  | TTAAAAAAAAAAACCCACCC | chr4:102501735-102501944 |
|  |  | TTTTAGTTATTGTTAATTTTTTT |  |
|  |  | GTTTTTTTTTTTTTTAGTYGGTA | chr4:102501700-102501949 |
|  |  | ATACCTCTCAACTACTATCAA |  |
|  |  | GAAAGAGAGAGAAGTGTATTA | chr4:102501348-102501640 |
|  |  | AAACCRAACCAAACCAATCAA |  |
|  |  | AGAAAGAGAGAGAAGTGTATTA | chr4:102501348-102501640 |
|  |  | RAAACCRAACCAAACCAATCAA |  |
|  |  | AATATAAATATAACTAAAACA | chr4:102500812-102501085 |
|  |  | TTTTGGTTTTTTAGTAGGGT |  |
|  |  | TAAAAACTTCCCTACCRACT | chr4:102501024-102501300 |
|  |  | GAGAGAGTATATAGATAGAT |  |
| *ESR1* | chr6:151807688-151808636 / 105 | GTGTATTTGGATAGTAGTAA | chr6:151808036-151808313 |
|  |  | TTCTCCAAATAATAAAACAC |  |
|  |  | TAATGTTAGGGTAAGGTAATAG | chr6:151807578-151807875 |
|  |  | RCAAAACAAAAAACTCAAAAAC |  |
|  |  | GATTATGATTTTTTATATTAAAG | chr6:151807915-151808209 |
|  |  | TAAAAAAAAACCCCCCAAAC |  |
|  |  | GGTTAGTAGGTGTTTTATTA | chr6:151808285-151808566 |
|  |  | RAAAAAACTTAACTCTAAAC |  |
|  |  | AGGGAGAAGGGAGAGTTTAG | chr6:151808429-151808690 |
|  |  | CACAAATAACTACTACTTTA |  |
|  |  | TTGTGTTTTTTTTTTAGGTGG | chr6:151807826-151808058 |
|  |  | AACTTACTACTATCCAAATAC |  |
|  |  | TATCAAAACAAAACAACAATCCC | chr6:151807581-151807872 |
|  |  | GGGTAGAAGGTTTAGAAATT |  |
|  |  | CTATACTCTTTTTCCAAATAAC | chr6:151807826-151808058 |
|  |  | GGTTTGTTGTTGTTTAGGTA |  |
|  |  | AATCCAAAAAAACRAACTAAAAC | chr6:151807963-151808214 |
|  |  | TTGAGTGGGGGGAAATTTTT |  |
|  |  | AAAACCTAAAAAACTACRAAAAC | chr6:151808440-151808606 |
|  |  | GGTAAGTGGGTGGAGAGTAT |  |
|  |  | AAATTTCCCCCCACTCAACA | chr6:151808197-151808457 |
|  |  | YGTAGTTTTTTAGGTTTTTTTT |  |
| *CDKN2A* | chr9:21974580-21975307 / 63 | AGGAGGTTTGTGATTATAAA | chr9:21974522-21974813 |
|  |  | AAAAAACAACATAAAACCTTC |  |
|  |  | TGGGTTTTTTTTTATTTGTTTT | chr9:21975164-21975372 |
|  |  | AAAAACCATACTTTCCCTATAAC |  |
|  |  | GGAAGAGTTTTTTTTGATTTTGTT | chr9:21974945-21975171 |
|  |  | AAAACCCAATCCTCCTTCCTTA |  |
|  |  | GAAGGTTTTATGTTGTTTTTT | chr9:21974793-21975053 |
|  |  | RAAATTAATAACACCTCCTC |  |
|  |  | RAAAACTCCATACTACTCCC | chr9:21974793-21975053 |
|  |  | GGAGTTAATAGTATTTTTTTT |  |
|  |  | TATCCCTCAAATCCTCTAAA | chr9:21974965-21975168 |
|  |  | GTTTAGTTTTTTTTTTTTGTT |  |
|  |  | AATATAACACCCCTAAAATC | chr9:21975066-21975280 |
|  |  | GGATTTTTTTTTAATAGAGT |  |
|  |  | RCCAAAAAAAAATCTATAATTAC | chr9:21974516-21974683 |
|  |  | ATTTAGGTGGGTAGAGGGTTT |  |
| *H19* | chr11:1998336-1998633 / 27 | GGGTTTTTGGGGATTT | chr11:1998207-1998469 |
|  |  | CTTCAAACAAAAAAATAACC |  |
|  |  | GGAGATAGGGTTGAGTATTG | chr11:1998055-1998250 |
|  |  | TAACAAAAAAAAACCCTCTA |  |
|  |  | GGTGTTAGTTTTTTAGGTT | chr11:1998339-1998590 |
|  |  | AATCTCCACTCCACTCC |  |
|  |  | GGTTATTTTTTTGTTTGAAG | chr11:1998450-1998746 |
|  |  | TCTAAATCCCTTAACCCTAATA |  |
|  |  | CCATCCTAAAATTCTCCAAA | chr11:1998003-1998297 |
|  |  | GTATTTTATTTTTATTTTTTAGG |  |
|  |  | TCCAATTAACCRAACTTATACTAA | chr11:1998366-1998659 |
|  |  | TTTTTATTAAAGGTTAAGGT |  |
|  |  | TACTACTCCCTACCTACCAA | chr11:1998147-1998319 |
|  |  | YGGTATTTTTTTTTGTTTTTTAG |  |
| *IGF2* | chr11:2137722-2141254 / 330 | TTAGAGAATTTAGGGGTTTT | chr11:2139678-2139928 |
|  |  | CTACTAAAACAAATAACRACT |  |
|  |  | TTAAAACCTCAACCAAATCC | chr11:2139950-2140215 |
|  |  | GGGGTAATTATTATTTAGATA |  |
|  |  | AGGGAGTAGGAGTTTTATTAT | chr11:2134297-2134538 |
|  |  | TACACTTCAAAACTTCTCTCT |  |
|  |  | CAAATAAAAACTTAACCCTC | chr11:2134328-2134556 |
|  |  | GGGTTTATAGGATGTAAATGTAT |  |
|  |  | RTAATAAAACCACTCCCTTCTTT | chr11:2134169-2134387 |
|  |  | GGGAGAAGTTTTTTTGTATT |  |
|  |  | ATAGTAATGTTTAGTTGGAAGG | chr11:2134216-2134450 |
|  |  | ACCTACCCCATTTAAAAATA |  |
|  |  | TTGTTTTTAGAGAGTGGGAAA | chr11:2133478-2133768 |
|  |  | AAACCTAACCTAAAAACAAAA |  |
|  |  | TTTATAGTATAGAGAGAGTYGTGTTAG | chr11:2133674-2133927 |
|  |  | AACTTATACCCATTTTTCAT |  |
|  |  | ATTTCTCTATCTCTAAAAAATAA | chr11:2133472-2133770 |
|  |  | TAGGGTTTGGTTTGAGGGTA |  |
|  |  | RAACCTACCTAAAAATCCCA | chr11:2133658-2133933 |
|  |  | ATTAAGAGTTTGTGTTTATT |  |
|  |  | TAAGTTTAGTTAAGGGGAAG | chr11:2140393-2140612 |
|  |  | AAAAAATCCCAAAATAAAACRAA |  |
|  |  | TTTGGAGTTTTAGTTAGATTTYGA | chr11:2139949-2140108 |
|  |  | CTAATCCTCTCTCCTCTAAC |  |
|  |  | GAATTTGGGTTAGGTTTGGA | chr11:2140050-2140307 |
|  |  | CTCTCCAAAAAATAATTTCC |  |
| *APOA5* | chr11:116790319-116790694 / 34 | TATGGGTGGAAGAGTTTTTT | chr11:116790648-116790823 |
|  |  | CTAATAAAACAAATAACCCT |  |
|  |  | GTYGGTTTTTTTTTTAGTTTTAG | chr11:116790416-116790666 |
|  |  | AAAAACTCTTCCACCCATAC |  |
|  |  | TGATGTTTTTTTATAGGTTA | chr11:116790175-116790453 |
|  |  | AAACCTTTACAAACACTAAA |  |
|  |  | TTACCACTATCTATTTATTA | chr11:116790225-116790489 |
|  |  | AGTAGAATTTGGATTAGTTG |  |
|  |  | TCCTCAATCCCAATACCTAC | chr11:116790426-116790676 |
|  |  | GGTYGTTTTAAAGAGTTTTTTTA |  |

* R - A or G

** Y - C or T

**Figure S1.** Map of the polluted region where exposed subjects are living (Kapan city, Artsvanik and Syunik villages).

**
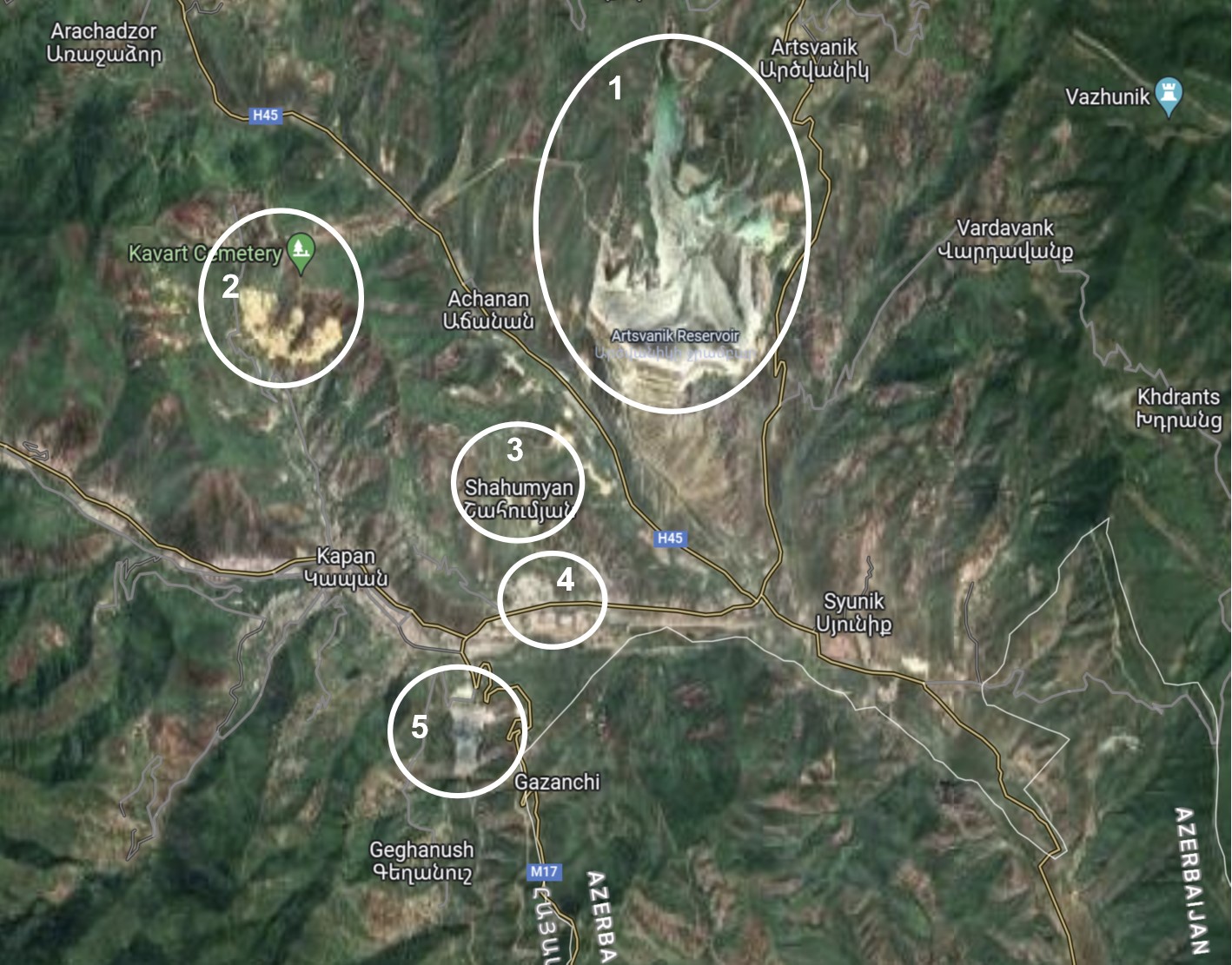
**

*White circles indicate locations of mining infrastructures: 1: Artsvanik tailing dump; 2: abandoned mining site; 3: Shahumyan mine; 4: processing plant; 5: Geghanush tailing dump (adapted from O. Belyaeva et al. [25]).*

**Figure S2**. Correlation matrix of chemical elements for exposed group


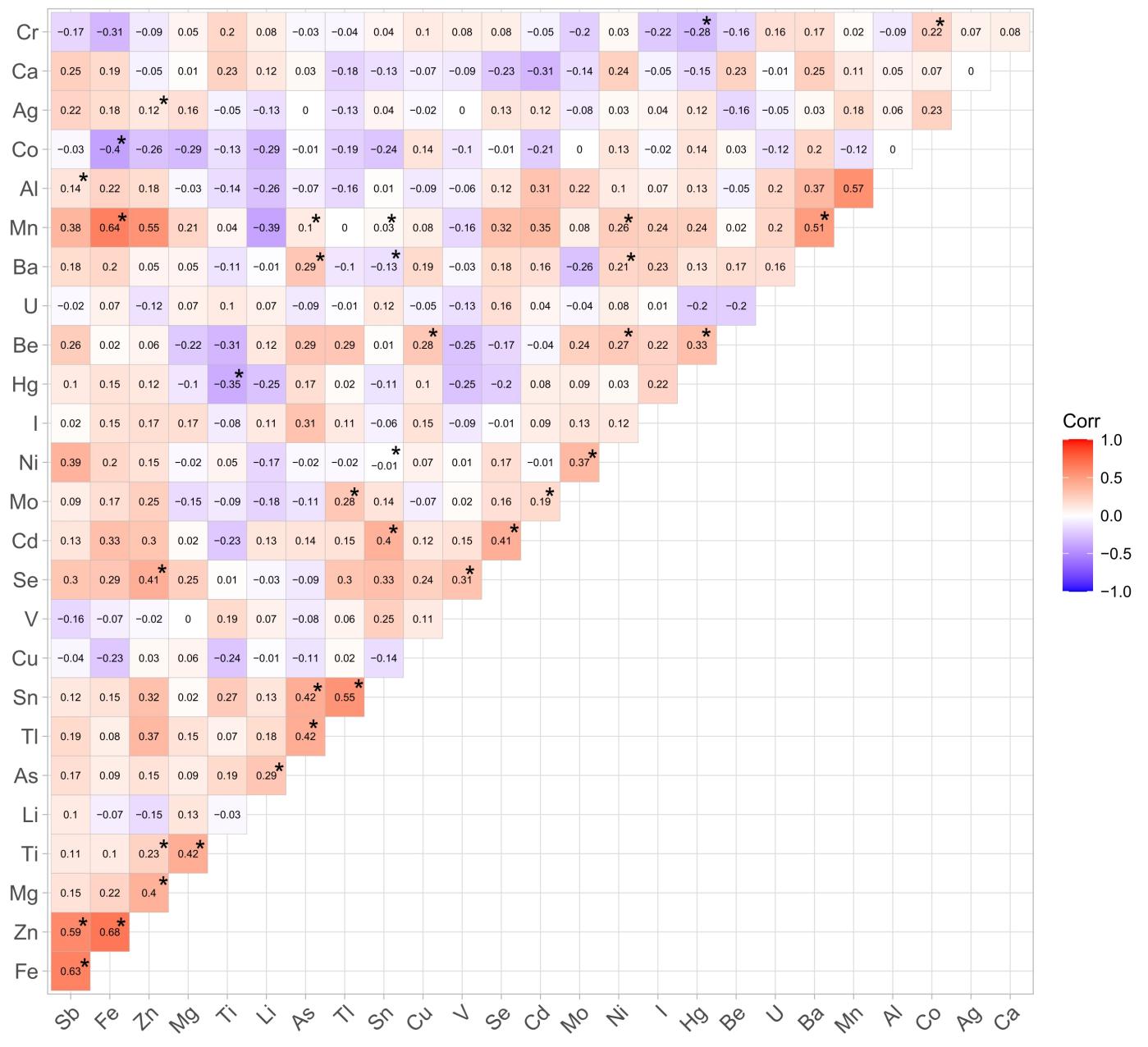


*A correlation matrix shows the correlation coefficients between metal plasma levels in the exposed group. Asterisk symbol (*) indicates significant correlations (p<0.05).*

**Figure S3**. Correlation matrix of chemical elements for unexposed group


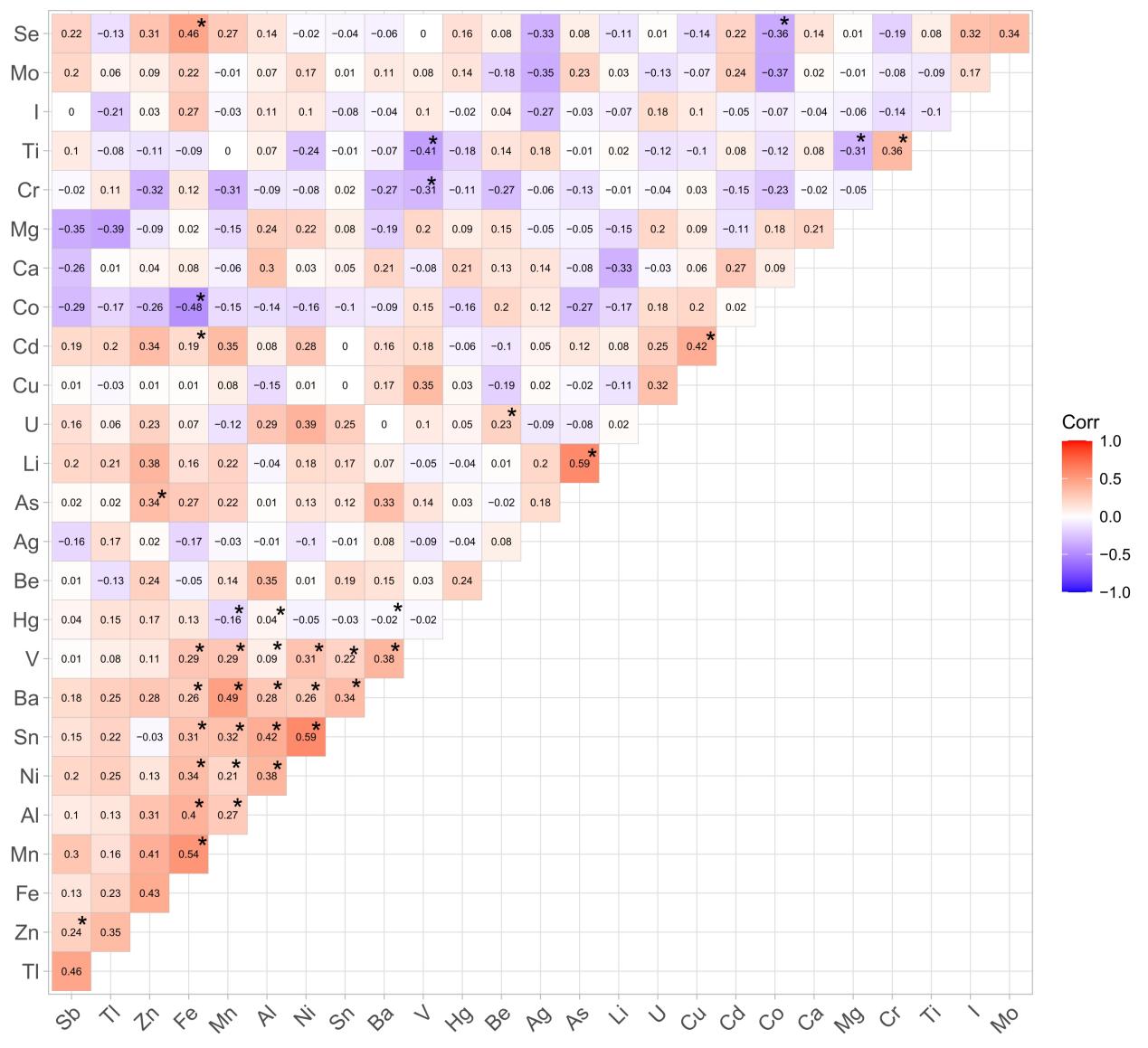


*A correlation matrix describes the strength of correlation between metal plasma levels in the unexposed group by coefficients. Asterisk symbol (*) indicates significant correlations (p<0.05).*

**Figure S4.** Correlation matrix of metal plasma levels and methylation levels of CpGs associated with metal exposure.

**
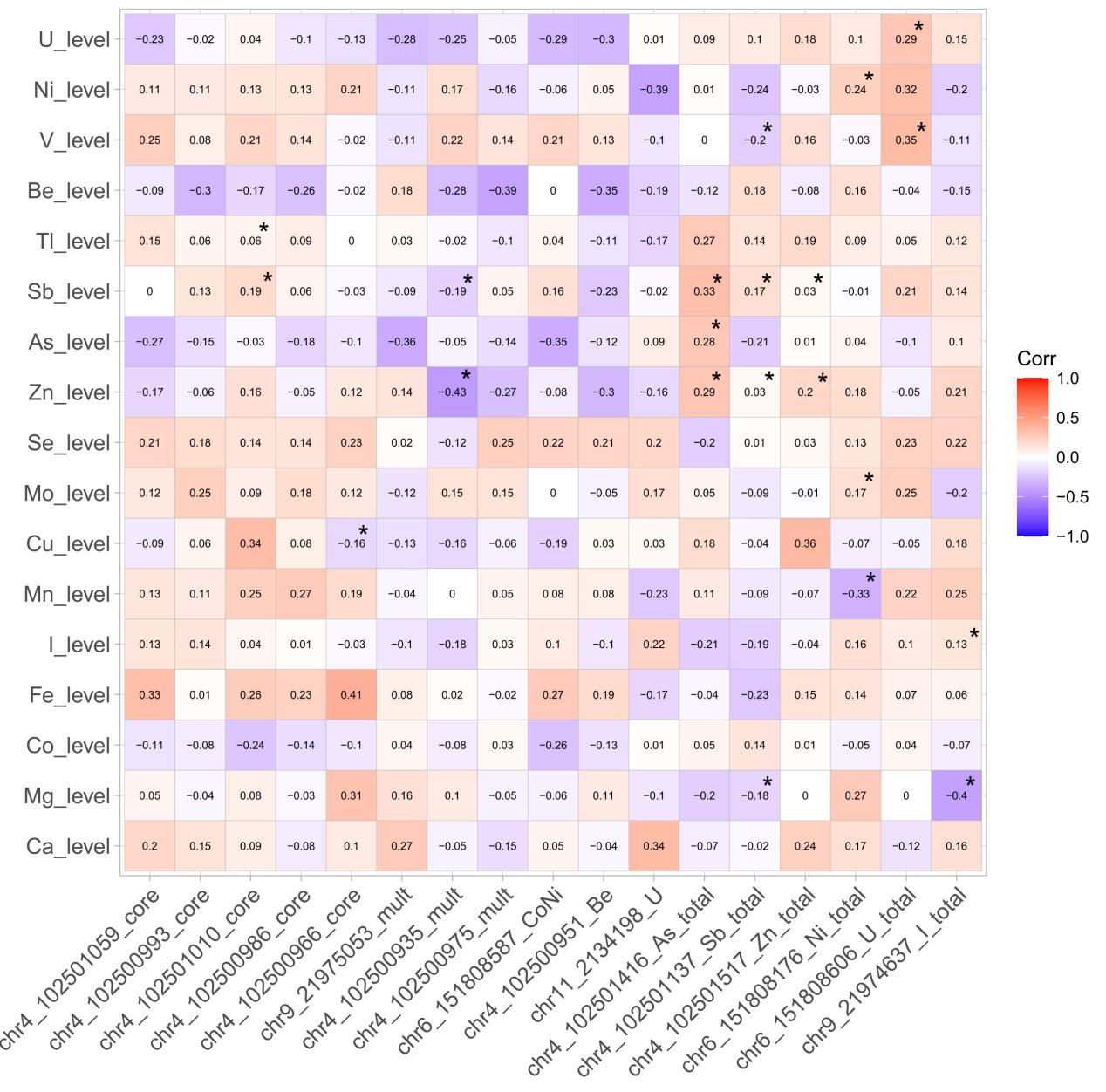
**

*A correlation matrix shows the correlation coefficients between metal plasma levels and methylation levels of CpGs associated with metal exposure. Asterisk symbol (*) indicates significant correlations (p<0.05).*

| Element (ppb) | mean  (percentile, P_2.5_-P_97.5_)  (female) | mean  (percentile, P_2.5_-P_97.5_)  (male) | mean  (percentile, P_2.5_-P_97.5_)  (all sexes) |
| --- | --- | --- | --- |
| Ca | 108x10^3^ (94x10^3^-119x10^3^) | 110x10^3^ (94x10^3^-119x10^3^) | 109x10^3^ (94x10^3^-119x10^3^) |
| Mg | 22.6x10^3^ (19.0x10^3^-25.0x10^3^) | 22.2x10^3^ (20.0x10^3^-24.6x10^3^) | 22.4x10^3^ (19.0x10^3^-25.0x10^3^) |
| Co | 0.684 (0.366-0.001) | 0.449 (0.302- 0.884) | 0.582 (0.303 - 0.001) |
| Fe | 1945 (1177-3173) | 2729 (2002-4050) | 2287 (1206-3747) |
| I | 75 (60-86) | 78 (60-113) | 77 (59-93) |
| Mn | 3 (2-3) | 4 (2-17) | 3 (2-7) |
| Cu | 1205 (883-1540) | 1127 (877-1502) | 1171 (865-1550) |
| Mo | 1 (0.74-3) | 2 (1-3) | 2 (0.79-3) |
| Se | 130 (114-150) | 139 (124-153) | 134 (116-153) |
| Cr | 3 (2-3) | 3 (2-3) | 3 (2-3) |
| Zn | 1570 (1255-2458) | 1614 (1394-1818) | 1589 (1260-1919) |
| Cd | 0.077 (0.037-0.151) | 0.116 (0.038-0.421) | 0.098 (0.037-0.343) |
| As | 2 (2-3) | 2 (2-3) | 2 (2-3) |
| Hg | 0.367 (0.300-0.500) | 0.475 (0.318-0.683) | 0.429 (0.300-0.668) |
| Sb | 8(7-16) | 8 (7-9) | 8 (7-10) |
| Tl | 0.027 (0.007-0.063) | 0.047 (0.021-0.131) | 0.036 (0.007-0.073) |
| Al | 24 (19-33) | 39 (24-133) | 31 (19-52) |
| Ba | 2 (1-3) | 4 (1-16) | 3 (1-11) |
| Be | 0.163 (0.100-0.300) | 0.179 (0.100-0.435) | 0.170 (0.100-0.340) |
| V | 4 (4-5) | 4 (4-5) | 4 (4-5) |
| Bi | < 0.002 (LOD) | | |
| Au | < 0.06 (LOD) | | |
| Li | 4 (2-9) | 5 (3-9) | 4 (2-10) |
| Ni | 8 (7-10) | 9 (8-12) | 9 (7-11) |
| Sn | 0.146 (0.100-0.348) | 0.224 (0.100-0.680) | 0.180 (0.100-0.515) |
| Pt | < 0.003 (LOD) | | |
| Ag | 0.17 (0.10-0.51) | 0.10 (0.10-0.10) | 0.15 (0.10-0.47) |
| Ti | 9 (8-9) | 9 (8-9) | 9 (8-9) |
| U | 0.017 (0.011-0.025) | 0.027 (0.012-0.113) | 0.022 (0.011-0.060) |

**Table S3**. Reference levels of 26 chemical elements in human blood plasma for both genders in Armenian population.
